# Epidemiologic and clinicopathologic features of 19,352 jaw cysts: a single-center retrospective study

**DOI:** 10.1186/s12903-026-07776-y

**Published:** 2026-02-02

**Authors:** Katsutoshi Kokubun, Yoshihiko Akashi, Kei Nakajima, Kei Yamamoto, Akira Watanabe, Akira Katakura, Kenichi Matsuzaka

**Affiliations:** 1 Department of Pathology, Tokyo Dental College, 2-9-18 Kandamisaki-cho, Chiyoda-ku, Tokyo, 101-0061 Japan; 2 Department of Oral and Maxillofacial Surgery, Tokyo Dental College, 2-9-18 Kandamisaki-cho, Chiyoda-ku, Tokyo, 101-0061 Japan; 3 Department of Oral Pathobiological Science and Surgery, Tokyo Dental College, 2-9-18 Kandamisaki-cho, Chiyoda-ku, Tokyo, 101-0061 Japan

**Keywords:** Epidemiology, Jaw cysts, Maxillofacial lesions, Odontogenic cysts, Oral pathology

## Abstract

**Background:**

Jaw cysts are a diverse group of intraosseous lesions commonly encountered in oral and maxillofacial pathologies. Several studies have addressed their distribution and clinicopathological features; however, further large-scale analyses using standardized classification systems may enhance cross-regional comparability and diagnostic consistency. We aimed to evaluate the demographic and anatomical characteristics of jaw cysts over an almost 50-year period.

**Methods:**

We retrospectively reviewed 19,352 histologically confirmed jaw cysts diagnosed between 1975 and 2024. Each case was reclassified according to the 2022 WHO classifications for head and neck tumors. Patient age, sex, cyst type, and anatomical location data were collected and descriptively analyzed to identify trends across different cyst categories.

**Results:**

Odontogenic cysts comprised the majority of cases. Radicular cysts were the most common, followed by dentigerous cysts and odontogenic keratocysts. Non-odontogenic cysts mainly consisted of surgical ciliated cysts and nasopalatine duct cysts. Bone cysts (simple bone cysts and aneurysmal bone cysts), which lack an epithelial lining and were therefore analyzed separately, were infrequent. A male predominance was observed overall, with sex- and age-related patterns differing according to cyst type. Mandibular involvement was more common than maxillary involvement, and several cysts showed specific anatomical predilections. These findings highlight distinct demographic and anatomical characteristics across cyst categories.

**Conclusions:**

This large retrospective study provides a detailed epidemiological profile of jaw cysts. The findings revealed distinct patterns according to cyst type, age, sex, and anatomical site, providing a valuable reference for diagnostic refinement and future comparative studies of oral and maxillofacial pathology.

## Background

Cystic lesions of the jaw encompass a diverse group of intraosseous conditions characterized with cavity formation, typically lined with epithelium, and containing fluid or semi-solid material. Most of these lesions originate from odontogenic epithelium and are commonly encountered in oral and maxillofacial clinical practice. They include both inflammatory cysts, such as radicular cysts secondary to pulp necrosis, and developmental cysts, such as dentigerous cysts and odontogenic keratocysts, which arise from dental lamina or reduced enamel epithelium in the absence of infection [1–3]. In addition to these epithelial-lined cysts, the jaw also harbors non-epithelialized lesions such as simple bone cysts and aneurysmal bone cysts, which lack an epithelial lining and are therefore categorized separately in epidemiologic analyses.

The classification of jaw cysts has evolved with advancements in histopathology and molecular genetics. For example, in the 2005 WHO classification, an odontogenic keratocyst was reclassified as a neoplasm (keratocystic odontogenic tumor) owing to its aggressive behavior and association with *PTCH1* gene mutations. However, it was redefined as a cyst in the 2017 and 2022 WHO classifications based on its cystic structure and lack of some neoplastic features [1, 4–8]. Similar revisions have affected the diagnostic criteria and nomenclature of other entities, including the calcifying odontogenic cyst and the glandular odontogenic cyst, leading to variations in incidence reporting across studies [7, 9].

Despite their high clinical prevalence, the epidemiological features of jaw cysts have not been comprehensively characterized, particularly in large-scale studies using standardized classification systems. A major limitation of the literature is heterogeneity in the diagnostic terminology, inclusion criteria, and biopsy submission protocols. Some studies include incisional and excisional biopsies, whereas others limit the analyses to surgically removed specimens. Moreover, changes in WHO classifications over time complicate longitudinal comparisons [10–12]. Regional differences in surgical practice also influence lesion frequency, as exemplified by surgical ciliated cysts, which are underrepresented in Western series but relatively common in East Asia owing to the prevalence of radical maxillary sinus surgery [13, 14].

Multicenter studies have generated valuable epidemiological data [1] ; however, inconsistencies in histopathological interpretations across institutions pose challenges. Conversely, single-center studies benefit from diagnostic consistency and are well suited for retrospective analyses when using a uniform classification framework, such as the 2022 WHO classification system.

This study aimed to provide a comprehensive, single-center, epidemiological analysis of jaw cysts over an extended period. Using standardized criteria based on the latest WHO classification, we examined the frequency, demographic distribution, and anatomical location of various cyst types. Through comparing our results with international data, we also aimed to clarify diagnostic trends, identify region-specific patterns, and provide reliable findings for future multicenter investigations and classification updates.

## Methods

In this retrospective study, we reviewed 19,352 jaw cysts diagnosed at Tokyo Dental College Hospital between 1975 and 2024 based on clinical records, including data concerning patient age, sex, and anatomical lesion location. Excisional and incisional biopsy specimens were included. When necessary, cases were reclassified according to the WHO Classification of Head and Neck Tumours 5th Edition. Diagnoses were confirmed through reviewing the histopathological reports, including histological and immunohistochemical descriptions. To ensure diagnostic accuracy, two experienced oral pathologists independently performed the assessments. In cases of disagreement or diagnostic uncertainty, a third expert was consulted, and a consensus diagnosis was reached. The following variables were included in the analysis, as described in previous studies: patient demographics (age and sex), anatomical site, and histopathological diagnosis.

Cysts were classified into four major groups [4]:

- Odontogenic cysts: radicular cyst, dentigerous cyst, odontogenic keratocyst, inflammatory collateral cysts, calcifying odontogenic cyst, orthokeratinized odontogenic cyst, glandular odontogenic cyst, lateral periodontal cyst and botryoid odontogenic cyst, gingival cyst.
- Non-odontogenic cysts: surgical ciliated cyst, nasopalatine duct cyst.
- Bone cysts: simple bone cyst, aneurysmal bone cyst (analyzed separately from odontogenic and non-odontogenic epithelial-lined cysts owing to the lack of an epithelial lining).
- Cyst: not otherwise specified (NOS).

Anatomical sites were categorized as follows:

- Maxilla: anterior, posterior, anterior–posterior, and NOS.
- Mandible: anterior, posterior, anterior–posterior, angle–ramus, and NOS.

Data analyses were performed using IBM SPSS Statistics for Mac, version 30.0 software. To assess sex differences across diagnostic groups, a chi-square test was applied, and a *p*-value < 0.05 was considered statistically significant. Welch’s *t*-test was used to compare the mean age between diagnostic categories and between sexes, with significance set at *p* < 0.05. Cross-tabulation analyses were conducted to determine the association between cyst type and anatomical location, and a chi-square test was used to determine the statistical significance of the distribution of cysts across different sites. For all comparisons described above, corresponding *p*-values are reported in the Results section.

## Results

In total, 19,352 jaw cysts were included in this study. Odontogenic cysts were the most prevalent (*n* = 16,359; 84.53%), followed by non-odontogenic cysts (*n* = 1,922; 9.93%) and bone cysts (*n* = 77; 0.40%) (Table 1).

**Table 1:** Frequency and percentage of jaw cyst diagnoses

Pathological diagnosis		Number	% total
Odontogenic cysts		16,359	84.53
	Radicular cyst	8628	44.58
	Dentigerous cyst	6273	32.42
	Odontogenic keratocyst	1164	6.01
	Inflammatory collateral cyst	165	0.85
	Calcifying odontogenic cyst	61	0.32
	Orthokeratinized odontogenic cyst	25	0.13
	Glandular odontogenic cyst	24	0.12
	Lateral periodontal cyst and botryoid odontogenic cyst	13	0.07
	Gingival cyst	6	0.03
Non-odontogenic cysts		1922	9.93
	Surgical ciliated cyst	1422	7.35
	Nasopalatine duct cyst	500	2.58
Bone cysts		77	0.40
	Simple bone cyst	63	0.33
	Aneurysmal bone cyst	14	0.07
Cyst, NOS		994	5.14
Total		19,352	100.00

Statistically significant differences (*p* < 0.05) are indicated later within the Results section text

*NOS* Not otherwise specified.

The most frequently diagnosed lesions were radicular cysts (*n* = 8,628; 44.58%), dentigerous cysts (*n* = 6,273; 32.42%), and surgical ciliated cysts (*n* = 1,422; 7.35%). Together, these three entities accounted for 84.35% of all cases. Other notable diagnoses included odontogenic keratocysts (*n* = 1,164; 6.01%), nasopalatine duct cysts (*n* = 500; 2.58%), and simple bone cysts (*n* = 63; 0.33%).

**Fig. 1:**
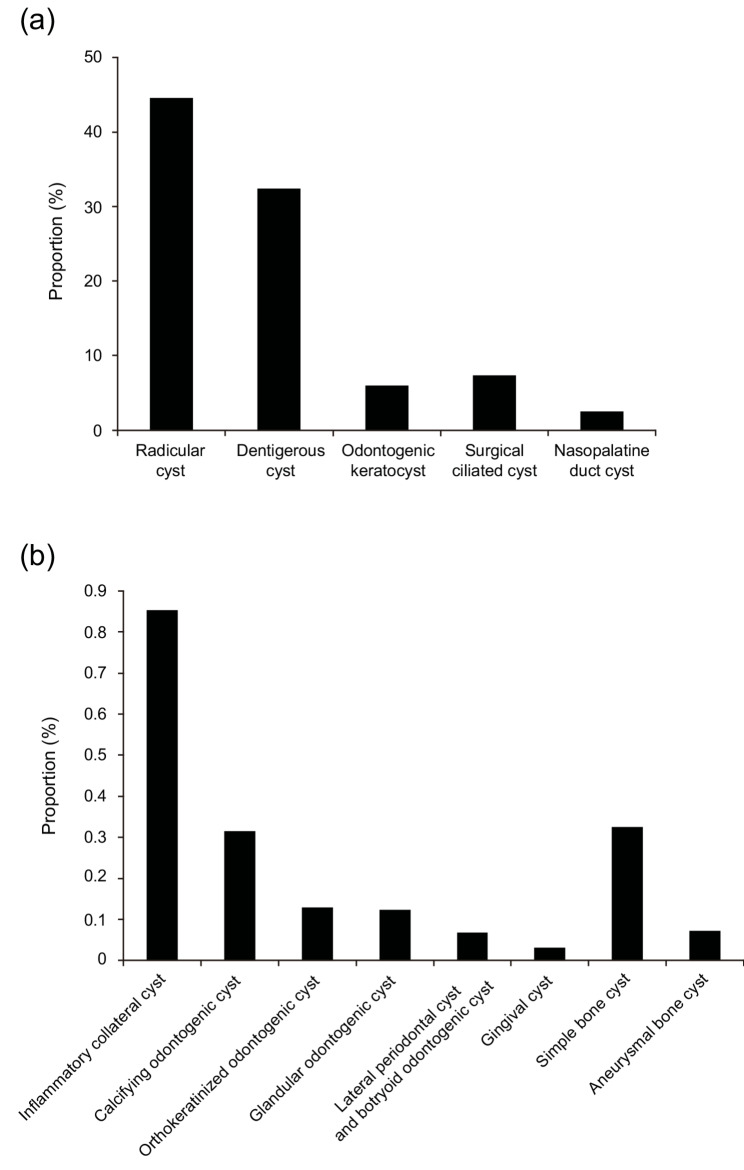
**a** Relative frequency of major jaw cysts in this study, (**b**) Relative frequency of rare jaw cysts in this study

A graphical summary of the relative frequency of all cyst categories included in this study is presented in Fig. 1. Within the odontogenic cyst group (*n* = 16,359), radicular cysts were the most frequent (*n* = 8,628; 52.74%), followed by dentigerous cysts (*n* = 6,273; 38.35%) and odontogenic keratocysts (*n* = 1,164; 7.11%). The remaining odontogenic cysts accounted for < 1% of the total cysts. In the non-odontogenic group (*n* = 1,922), surgical ciliated cysts predominated (*n* = 1,422; 73.99%), with nasopalatine duct cysts comprising the remainder (*n* = 500; 26.01%). Among bone cysts (*n* = 77), simple bone cysts were most common (*n* = 63; 81.82%), followed by aneurysmal bone cysts (*n* = 14; 18.18%).

Of the 19,352 study patients, 11,549 (59.67%) were males and 7,803 (40.33%) were females, yielding an overall male-to-female ratio of 1.48:1 (Table 2).

**Table 2:** Sex distribution of jaw cysts according to diagnostic category

Pathological diagnosis		Males	Females	M: F ratio
Odontogenic cysts		9720	6639	1.46:1
	Radicular cyst	4849	3779	1.28:1
	Dentigerous cyst	3985	2288	1.74:1
	Odontogenic keratocyst	707	457	1.55:1
	Inflammatory collateral cyst	99	66	1.5:1
	Calcifying odontogenic cyst	35	26	1.35:1
	Orthokeratinized odontogenic cyst	19	6	3.17:1
	Glandular odontogenic cyst	17	7	2.43:1
	Lateral periodontal cyst and botryoid odontogenic cyst	6	7	0.86:1
	Gingival cyst	3	3	1:1
Non-odontogenic cysts		1143	779	1.47:1
	Surgical ciliated cyst	837	585	1.43:1
	Nasopalatine duct cyst	306	194	1.58:1
Bone cysts		35	42	0.83:1
	Simple bone cyst	25	38	0.66:1
	Aneurysmal bone cyst	10	4	2.5:1
Cyst, NOS		651	343	1.9:1
Total		11,549	7803	1.48:1

Statistically significant differences (*p* < 0.05) are indicated later within the Results section text

*NOS* Not otherwise specified

**Fig. 2:**
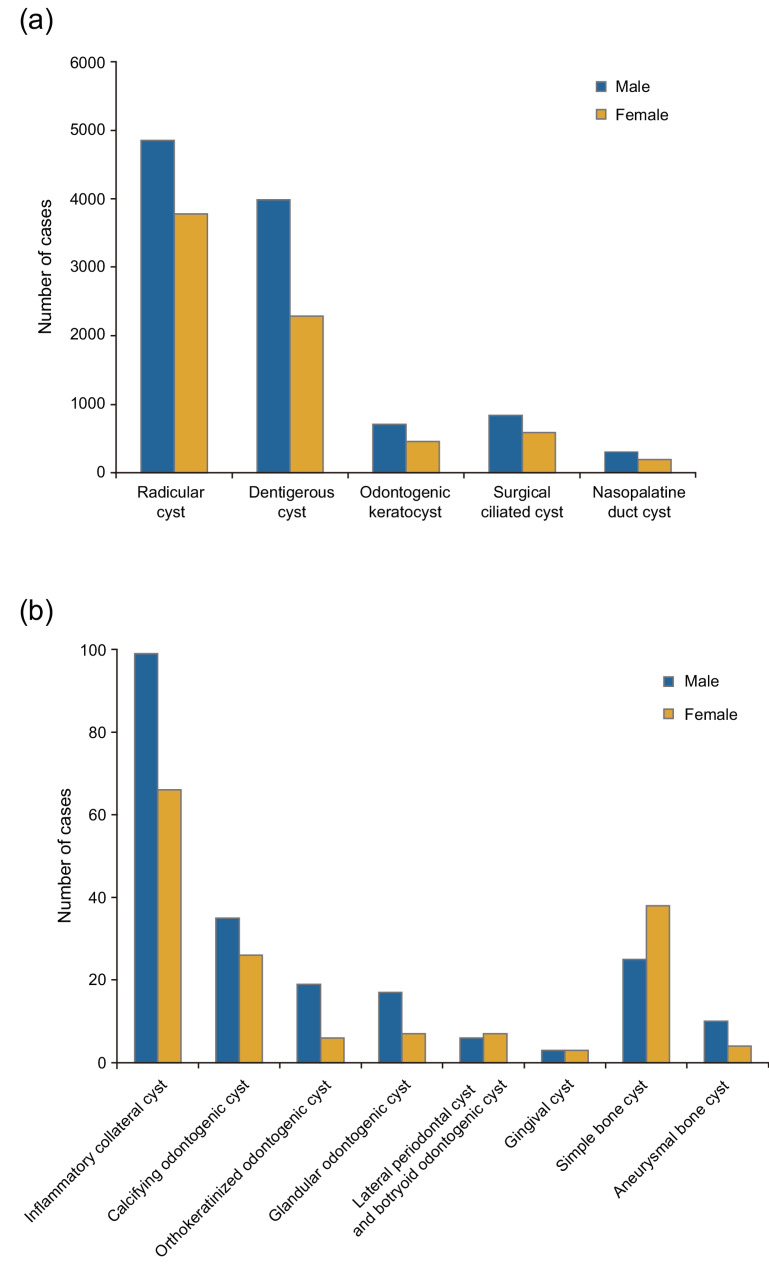
**a** Sex distribution of major jaw cysts, (**b**) Sex distribution of rare jaw cysts

The male-to-female ratios were 1.46:1 for odontogenic cysts, 1.47:1 for non-odontogenic cysts, and 0.83:1 for bone cysts. Sex distribution patterns across all cyst categories included in this study are shown in Fig. 2. Within the odontogenic cyst group, a significant male predominance was observed in radicular cysts (males, *n* = 4,849; females, *n* = 3,779; ratio, 1.28:1; *p* < 0.001) and dentigerous cysts (males, *n* = 3,985; females, *n* = 2,288; ratio, 1.74:1; *p* < 0.001). In the non-odontogenic group, surgical ciliated cysts exhibited a male-to-female ratio of 1.43:1 (males, *n* = 837; females, *n* = 585), and nasopalatine duct cysts exhibited a ratio of 1.58:1 (males, *n* = 306; females, *n* = 194). In the bone cyst group (*n* = 77), simple bone cysts were significantly more common in females (males, *n* = 25; females, *n* = 38; ratio, 0.66:1; *p* = 0.001).

The overall mean age at the diagnosis of jaw cysts was 42.74 years (standard deviation [SD], 17.02) (Table 3).

**Table 3:** Mean age and SD of patients with jaw cysts according to diagnosis and sex

Pathological diagnosis		Mean age ± SD of all patients			Mean age ± SD of male patients			Mean age ± SD of female patients		
Odontogenic cysts		42.65	±	17.16	42.70	±	16.52	42.57	±	18.06
	Radicular cyst	45.52	±	16.00	44.92	±	15.19	46.30	±	16.96
	Dentigerous cyst	39.63	±	17.60	40.50	±	17.13	38.10	±	18.29
	Odontogenic keratocyst	38.64	±	18.85	40.51	±	19.06	35.74	±	18.19
	Inflammatory collateral cyst	41.82	±	15.69	43.23	±	15.12	39.70	±	16.51
	Calcifying odontogenic cyst	25.56	±	20.44	24.77	±	19.57	26.62	±	22.29
	Orthokeratinized odontogenic cyst	41.68	±	17.24	42.89	±	18.91	37.83	±	13.26
	Glandular odontogenic cyst	42.25	±	16.08	46.35	±	15.86	32.29	±	14.13
	Lateral periodontal cyst and botryoid odontogenic cyst	39.15	±	19.26	44.00	±	25.16	35.00	±	15.25
	Gingival cyst	53.33	±	11.44	58.00	±	12.12	48.67	±	13.43
Non-odontogenic cysts		47.65	±	13.62	47.04	±	13.29	48.54	±	14.05
	Surgical ciliated cyst	49.24	±	12.29	48.56	±	11.94	50.22	±	12.73
	Nasopalatine duct cyst	43.11	±	15.98	42.89	±	15.73	43.46	±	16.45
Bone cysts		27.70	±	15.59	25.54	±	15.28	29.50	±	15.98
	Simple bone cyst	26.71	±	15.06	22.24	±	12.39	29.66	±	16.25
	Aneurysmal bone cyst	32.14	±	17.07	33.80	±	19.15	28.00	±	15.12
Cyst, NOS		35.92	±	17.54	36.04	±	17.51	35.68	±	17.66
Total		42.74	±	17.02	42.70	±	16.44	42.79	±	17.85

Statistically significant differences (*p* < 0.05) are indicated later within the Results section text.

*NOS* Not otherwise specified, *SD* Standard deviation.

According to diagnostic group, the mean age was 42.65 years (SD, 17.16) for odontogenic cysts, 47.65 years (SD, 13.62) for non-odontogenic cysts, and 27.70 years (SD, 15.59) for bone cysts. Among individual cyst types, surgical ciliated cysts were associated with the highest mean age (49.24 years; SD, 12.29), followed by radicular cysts (45.52 years; SD, 16.00) and nasopalatine duct cysts (43.11 years; SD, 15.98). Dentigerous cysts (39.63 years; SD, 17.60) and odontogenic keratocysts (38.64 years; SD, 18.85) were more common at younger ages, while calcifying odontogenic cysts occurred at the youngest mean age (25.56 years; SD, 20.44). Statistically significant sex-related differences in the mean age were identified among the five cyst types. Women with radicular cysts were slightly older than males (46.30 vs. 44.92 years, respectively; *p* < 0.001). Regarding dentigerous cysts, males were older than females (40.50 vs. 38.10 years, respectively; *p* < 0.001), as was the case for odontogenic keratocysts (40.51 vs. 35.74 years, respectively; *p* < 0.001). Among patients with surgical ciliated cysts, females had a slightly higher mean age than males (50.22 vs. 48.56 years, respectively; *p* = 0.013). The largest sex-based age gap was observed in relation to simple bone cysts, with females averaging 29.66 years and males averaging 22.24 years (*p* = 0.045).

The frequency of jaw cysts peaked in patients in their 40 s (*n* = 4,170), followed by those in their 30 s (*n* = 3,720) and 50 s (*n* = 3,588), collectively accounting for 59.3% of all cases (Table 4).

**Table 4:** Age distribution of jaw cysts according to diagnostic category

Pathological diagnosis		Age group (years)									
		0–9	10–19	20–29	30–39	40–49	50–59	60–69	70–79	80–89	90–99
Odontogenic cysts		261	1381	2250	3169	3493	2952	1835	817	186	15
	Radicular cyst	34	330	1154	1704	1940	1704	1108	517	126	11
	Dentigerous cyst	213	825	747	1233	1345	1065	584	213	44	4
	Odontogenic keratocyst	6	191	279	189	151	144	121	71	12	0
	Inflammatory collateral cysts	1	3	44	27	40	28	12	9	1	0
	Calcifying odontogenic cyst	6	28	13	3	2	0	5	2	2	0
	Orthokeratinized odontogenic cyst	0	1	7	5	4	3	3	2	0	0
	Glandular odontogenic cyst	0	2	3	6	5	6	0	1	1	0
	Lateral periodontal cyst and botryoid odontogenic cyst	1	1	3	1	4	1	1	1	0	0
	Gingival cyst	0	0	0	1	2	1	1	1	0	0
Non-odontogenic cysts		7	21	153	364	509	485	279	90	13	1
	Surgical ciliated cyst	2	2	64	260	406	393	219	68	8	0
	Nasopalatine duct cyst	5	19	89	104	103	92	60	22	5	1
Bone cysts		2	35	12	6	15	4	2	1	0	0
	Simple bone cyst	0	32	11	4	11	3	1	1	0	0
	Aneurysmal bone cyst	2	3	1	2	4	1	1	0	0	0
Cyst, NOS		45	141	224	181	153	147	67	32	2	2
Total		315	1578	2639	3720	4170	3588	2183	940	201	18

*NOS* Not otherwise specified

Odontogenic cysts were most common in individuals aged 30–59 years, with a broad peak in mid-adulthood. Non-odontogenic cysts followed a similar age distribution, peaking in patients in their 40 s and 50s. In contrast, bone cysts occurred predominantly in younger age groups, particularly among patients in their teens and 20s. Age distribution patterns varied according to cyst type. Radicular cysts were the most prevalent lesions across all age groups from the 20 s onward, peaking in the 40–49 (*n* = 1,940; 22.48%), 30–39 (*n* = 1,704; 19.75%), and 50–59 (*n* = 1,704; 19.75%)-year age groups. Dentigerous cysts peaked in patients in their 40 s (*n* = 1,345; 21.44%) and 30 s (*n* = 1,233; 19.66%). Odontogenic keratocysts were most common in patients in their 20 s (*n* = 279; 23.97%), 10 s (*n* = 191; 16.41%), and 30 s (*n* = 189; 16.24%). Simple bone cysts and calcifying odontogenic cysts showed a predilection for patients in their 20 s, with 50.79% and 45.90% of the cases diagnosed in patients in their 10 s, respectively.

Anatomically, jaw cysts most frequently involved the posterior mandible and anterior maxilla (Table 5).

**Table 5:** Anatomical distribution of jaw cysts according to diagnosis and site

Pathological diagnosis		Maxilla					Mandible					
		Anterior maxilla	Posterior maxilla	Anterior-posterior maxilla	Maxilla, NOS	Total	Anterior mandible	Posterior mandible	Anterior-posterior mandible	Angle-ramus	Mandible, NOS	Total
Odontogenic cysts		4018	2142	315	175	6650	582	8628	243	97	159	9709
	Radicular cyst	3202	1503	233	145	5083	410	2922	126	1	86	3545
	Dentigerous cyst	668	439	47	27	1181	104	4875	32	35	46	5092
	Odontogenic keratocyst	117	190	29	3	339	51	615	76	59	24	825
	Inflammatory collateral cysts	2	6	1	0	9	1	154	0	1	0	156
	Calcifying odontogenic cyst	22	1	3	0	26	11	17	4	0	3	35
	Orthokeratinized odontogenic cyst	1	1	0	0	2	0	21	1	1	0	23
	Glandular odontogenic cyst	4	1	1	0	6	1	15	2	0	0	18
	Lateral periodontal cyst and botryoid odontogenic cyst	2	0	1	0	3	2	6	2	0	0	10
	Gingival cyst	0	1	0	0	1	2	3	0	0	0	5
Non-odontogenic cysts		500	1422	0	0	1922	0	0	0	0	0	0
	Surgical ciliated cyst	0	1422	0	0	1422	0	0	0	0	0	0
	Nasopalatine duct cyst	500	0	0	0	500	0	0	0	0	0	0
Bone cysts		0	4	0	0	4	20	41	4	4	4	73
	Simple bone cyst	0	2	0	0	2	19	33	4	3	2	61
	Aneurysmal bone cyst	0	2	0	0	2	1	8	0	1	2	12
Cyst, NOS		255	148	47	47	497	54	330	35	5	73	497
Total		4773	3716	362	222	9073	656	8999	282	106	236	10,279

Statistically significant differences (*p* < 0.05) are indicated later within the Results section text

*NOS* Not otherwise specified

Overall, mandibular involvement was more common than maxillary involvement, particularly at posterior sites. Odontogenic cysts were primarily located in the posterior mandible and anterior maxilla. Non-odontogenic cysts showed a marked maxillary preference. Bone cysts were observed almost exclusively in the mandible. Site-specific analyses revealed a distinct anatomical predilection. Radicular cysts were most frequently located in the anterior maxilla (*n* = 3,202; 37.11%), posterior mandible (*n* = 2,922; 33.87%), and posterior maxilla (*n* = 1,503; 17.42%; *p* < 0.001). Dentigerous cysts showed a strong preference for the posterior mandible (*n* = 4,875, 77.71%; *p* < 0.001). Odontogenic keratocysts were most often located in the posterior mandible (*n* = 615, 52.84%; *p* < 0.001). Certain lesions had highly specific anatomical distributions. Surgical ciliated cysts occurred exclusively in the posterior maxilla (*n* = 1,422) and nasopalatine duct cysts were restricted to the anterior maxilla (*n* = 500) (both, *p* < 0.001). Inflammatory collateral cysts and orthokeratinized odontogenic cysts occurred predominantly in the posterior mandible (*p* < 0.001). Simple bone cysts also showed a mandibular predilection, especially in the posterior (*n* = 33; 52.38%) and anterior (*n* = 19; 30.16%) regions (*p* < 0.001).

## Discussion

### Prevalence patterns

In this cohort of histologically confirmed jaw cysts, odontogenic cysts were the most prevalent category, followed by non-odontogenic cysts and bone cysts (Table 1). These proportions are consistent with earlier large-scale epidemiological studies conducted in Turkey [15], Greece [3], and Italy [7], where odontogenic cysts consistently constituted the majority of jaw cysts (Table 6).

**Table 6:** Comparison of the relative frequency of jaw cysts between this study and previously published series

Cyst type	Present study (Japan)		Dhanuthai et al. (Thailand, 2023)		Aquilanti et al. (Italy, 2021)		Tamiolakis et al. (Greece, 2019)		Lo Muzio et al. (Italy, 2017)		Tekkesin et al. (Turkey, 2012)		Jones et al. (UK., 2006)	
	cases	%	cases	%	cases	%	cases	%	cases	%	cases	%	cases	%
Total cases	19,352	100	25,628	100	2150	100	5294	100	2030	100	5088	100	7121	100
Radicular cyst	8649	44.7	10,536	41.1	1216	56.6	3035	57.3	865	42.6	2802	55.1	3724	52.3
Dentigerous cyst	6270	32.4	8307	32.4	502	23.3	766	14.5	806	39.7	529	10.4	1292	18.1
Odontogenic keratocyst	1161	6	3273	12.8	277	12.9	436	8.2			1048	20.6	828	11.6
Inflammatory collateral cyst	405	2.1	405	1.6	31	1.5	53	1	20	1.0	11	0.2	402	5.6
Calcifying odontogenic cyst	61	0.3	283	1.1	16	0.7	19	0.4			33	0.7	21	0.3
Orthokeratinized odontogenic cyst	25	0.1	115	0.5	26	1.2	15	0.3	9	0.4				
Glandular odontogenic cyst	24	0.1	260	1	25	1.2	19	0.4	14	0.7	23	0.5	11	0.2
Lateral periodontal cyst/Botryoid cyst	13	0.1	341	1.3	17	0.8	31	0.6	14	0.7	8	0.2	28	0.4
Gingival cyst	6	0.03	212	0.8	24	1.1	13	0.2	12	0.6			16	0.2
Surgical ciliated cyst	1422	7.3	27	0.1										
Nasopalatine duct cyst	500	2.6	723	2.8	16	0.7	129	2.4	38	1.9	66	1.3		
Simple bone cyst	63	0.3					9	0.2	4	0.2				
Aneurysmal bone cyst	14	0.1					11	0.2	6	0.3				

Four major lesion types—radicular cysts, dentigerous cysts, surgical ciliated cysts, and odontogenic keratocysts—comprised most cases in our series, similar to findings reported in previous epidemiologic studies. Radicular cysts were the most frequently diagnosed lesions, consistent with previous reports [2, 10, 16–18], but some studies have documented higher rates [3, 7, 15, 19–21]. Dentigerous cysts were the second most common, consistent with previous studies reporting a prevalence of approximately 30–32% across different populations [1, 11, 22, 23]. Surgical ciliated cysts accounted for 7.35% of all cases in this cohort, a markedly higher frequency than that reported in multinational datasets, such as 0.10% reported in 2024 by Dhanuthai et al. [1]. This discrepancy may reflect regional differences in the prevalence of maxillary sinus and orthognathic surgeries, which are predisposing factors for these lesions. In Japan, surgical ciliated cysts are relatively common, with studies reporting their detection in up to 20% of patients after radical maxillary sinus surgery [14]. Basu et al. [24] reported 23 cases of postoperative maxillary cysts and suggested that the true prevalence outside Japan may be underestimated. A study conducted in Brazil further supported this finding, reporting that surgical ciliated cysts comprised 5.2% of non-odontogenic cysts [25]. In this study, 6.01% of all cysts were odontogenic keratocysts. However, wide variations in prevalence, ranging from 1.3% to 27.36%, have been reported [1, 3, 7, 8, 10, 15, 17–21, 26–33]. Our findings are consistent with those of de Souza et al. [19] and Demirkol et al. [20]. Bone cysts were rare in this cohort, with most classified as either simple bone cysts or aneurysmal bone cysts, which is a distribution pattern similarly reported in other populations [2, 3, 34]. Owing to the lack an epithelial lining, these cysts were analyzed as a separate diagnostic category from odontogenic and non-odontogenic cysts. This distinction reflects their distinct pathogenesis and clinical behavior and is consistent with previous epidemiologic studies. Moreover, given their frequently asymptomatic nature, these lesions may be underdiagnosed in the absence of surgical intervention. Cysts that were NOS accounted for 5.14% of all jaw cysts. This designation was applied to cases in which the clinical information was insufficient to establish a definitive diagnosis. This result is consistent with Dhanuthai et al.’s [1] findings and highlights the importance of providing comprehensive clinical and radiographic information when submitting biopsy specimens for histopathological evaluation.

### Sex distribution

In this study, male patients accounted for a greater proportion of jaw cysts overall. This male predominance was observed in odontogenic and non-odontogenic cysts but was reversed in bone cysts, where females were slightly more frequently affected. This distribution pattern is consistent with several previous studies reporting a male predominance of jaw cysts [1–3, 7, 15, 20, 23, 31, 32]. Demirkol et al. [20] reported a male-to-female ratio of 1.4:1, Tamiolakis et al. [3] reported a male-to-female ratio of 1.6:1, and Lo Muzio et al. [2] reported a male-to-female ratio of 1.71:1. In contrast, de Souza et al. [19] observed a reverse trend, likely reflecting population-specific factors such as oral health behaviors and patterns of biopsy submission. Sex-specific analyses indicated statistically significant differences among the four cyst categories. Dentigerous cysts exhibited the most marked male predominance, followed by cysts NOS and radicular cysts. These findings are in line with earlier reports, including those by Meningaud et al. [31] (dentigerous cysts, 2.35:1; radicular cysts, 1.70:1), Aquilanti et al. [7] (dentigerous cysts, 1.9:1; radicular cysts, 1.69:1), Du et al. [35] (dentigerous cysts, 1.90:1; radicular cysts, 1.42:1), Avelar et al. [11] (dentigerous cysts, 1.74:1; radicular cysts, 1.28:1), and Lo Muzio et al. [2] (dentigerous cysts, 1.53:1; radicular cysts, 1.77:1). Dhanuthai et al. [1] also reported a male predominance in cysts NOS (1.32:1). In contrast, simple bone cysts were significantly more frequent in females, a finding echoed in other studies suggesting a possible female predominance in this subgroup [3, 33, 36]. The observed male predominance in relation to most odontogenic cysts may be attributed to several factors including greater exposure to dental trauma, delayed treatment-seeking behavior among men, and a higher prevalence of dental caries and endodontic infections [27]. Conversely, the higher incidence of simple bone cysts in females may indicate sex-related differences in bone metabolism or hormonal influences; however, further research is necessary to clarify these mechanisms.

### Age distribution

In this study, the mean age at diagnosis for patients with jaw cysts was 42.74 years, which is comparable to several previous reports [1, 3, 7], but younger mean ages, often within the third decade of life, have also been frequently reported [2, 15, 20, 23, 26, 32, 37]. Similarly, the mean age for odontogenic cysts was 42.65 years, consistent with certain earlier findings [1, 16, 31, 38], whereas several studies have reported a younger age at diagnosis [2, 10, 17–19, 26, 28, 32, 39, 40]. These discrepancies may reflect regional differences in access to dental care and clinical thresholds for biopsy. In this cohort, the frequency of jaw cysts peaked during the fourth decade of life, followed by the third and fifth decades. This age distribution pattern has been observed in various populations, including those investigated by Tamiolakis et al. [3] (peaks in those aged 40–59 years), Aquilanti et al. [7] (predominance in those aged 30–59 years), and Dhanuthai et al. [1] (a broad peak in middle age). Overall, 59.3% of the cases in this study occurred between the ages of 30 and 59 years. The patients with radicular cysts had a relatively high mean age at diagnosis, with the highest incidence observed between the third and fifth decades of life. While this finding aligns with several previous reports [1, 3, 7, 16], earlier peak ages typically in the second to fourth decades have been reported [2, 10, 15, 19, 20, 26, 32, 33]. These variations may be related to differences in the prevalence and management of dental caries across countries. The mean age of patients with dentigerous cysts was 39.63 years, with the highest frequency occurring in the third and fourth decades of life. While some studies report similar or slightly younger mean ages [1, 3, 16, 22, 33], several studies indicate a younger age of onset [2, 10, 15, 19, 20, 23, 26, 32, 39, 40], whereas others document diagnoses at older ages [7, 31]. This wide age distribution may reflect the asymptomatic nature of dentigerous cysts and delayed detection of lesions associated with impacted third molars, potentially leading to postponed surgical intervention [20]. The mean age of patients with odontogenic keratocysts was 38.64 years in this study. A similar age at diagnosis has been reported elsewhere [19, 26, 31, 39, 41, 42]. The highest number of diagnoses occurred in the second and third decades, consistent with other reported findings [19, 33, 43–46]. Surgical ciliated cysts were diagnosed at a later mean age (49.24 years), which is in line with their postsurgical origin and latency period before clinical manifestations [14]. Simple bone cysts and calcifying odontogenic cysts showed an earlier onset than the other cyst types. The mean age of patients with simple bone cysts was 26.71 years, with a peak frequency in the second decade; 50.79% of the cases were diagnosed in patients aged 10–19 years, and this finding is consistent with previous studies [2, 3]. The mean age for calcifying odontogenic cysts was 25.56 years, which accords with previously reported data [1], although some studies have documented a predilection for the fourth decade [3, 7, 15, 19]. The five cyst types exhibited statistically significant sex-based differences in mean age. For example, odontogenic keratocysts were diagnosed at a younger age in females (35.74 years) than in males (40.51 years; *p* < 0.001), a trend also observed in dentigerous cysts. Similar patterns have been reported [3], but the extent of the difference varies across studies.

### Anatomical distribution

The anatomical distribution of jaw cysts in this study revealed differences based on diagnostic group and cyst type. Overall, mandibular involvement was more common than maxillary involvement, particularly in posterior regions. Among the 19,352 cases analyzed, the posterior mandible and anterior maxilla accounted for a substantial proportion of all lesions. This pattern aligns with previously reported findings [1, 2, 7, 8, 10, 16, 18, 19, 23, 29]. Odontogenic cysts, the most prevalent diagnostic group, were primarily located in the posterior mandible and anterior maxilla. Radicular cysts showed a marked preference for the anterior maxilla followed by the posterior mandible. Anterior maxillary dominance has been widely reported [1–3, 10, 15, 18–20, 26–28, 30, 32, 33, 35, 40, 47], possibly because of the higher incidence of trauma and caries affecting the maxillary anterior teeth, which are often preserved despite periapical pathology [1, 30, 48]. Dentigerous cysts were most frequently located in the posterior mandible, reflecting a high prevalence of impacted mandibular third molars. This distribution has also been previously well documented [1–3, 7, 15, 18–20, 22, 26–28, 30, 32, 40, 47]. Odontogenic keratocysts also demonstrated a strong predilection for the posterior mandible, followed by the posterior and anterior maxilla. This anatomical pattern has consistently been observed in previous investigations [1, 3, 7, 15, 18, 19, 26–28, 30, 32, 40, 42]. Given the high recurrence rate and aggressive nature of odontogenic keratocysts in the posterior mandible, careful radiographic surveillance and surgical planning are essential. Non-odontogenic cysts exhibited greater anatomical specificity. All surgical ciliated cysts were located in the posterior maxilla, a distribution reflecting their origin from the maxillary sinus epithelium, typically following Caldwell–Luc procedures or orthognathic surgery [13, 14, 24, 25]. Nasopalatine duct cysts were located exclusively in the anterior maxilla, which is consistent with their embryological origin in the nasopalatine canal. This distribution has been consistently confirmed in several studies [1–3, 7, 15, 20, 27, 49, 50]. Other cyst types also demonstrated a strong anatomical preference. Inflammatory collateral cysts and orthokeratinized odontogenic cysts were predominantly located in the posterior mandible, consistent with previous reports [1, 3, 7]. Simple bone cysts occurred almost exclusively in the mandible and most commonly in the posterior and anterior regions. This mandibular predominance has been consistently documented in studies concerning bone cysts [2, 36, 51, 52]. These anatomical trends are clinically relevant for diagnosis and treatment planning. For instance, a radiolucent lesion in the anterior maxilla may suggest either a radicular cyst or a nasopalatine duct cyst, whereas a posterior maxillary lesion in a patient with a history of sinus surgery may indicate a surgical ciliated cyst. Conversely, posterior mandibular lesions in adolescents should raise suspicion of dentigerous cysts, odontogenic keratocysts, or simple bone cysts. These localization patterns, supported by multicenter analyses [1], have clear implications for preoperative evaluation. In summary, the anatomical distribution of jaw cysts in this cohort closely mirrors the findings of international studies and reflects the developmental origin and clinical behavior of these lesions. Recognizing site-specific tendencies facilitates more accurate radiological interpretation and histopathological correlation, especially in cases where incisional biopsies yield limited tissue samples.

### Future directions and clinical implications

From a broader perspective, this dataset can serve as a valuable reference for pathology education, diagnostic benchmarking, and public health planning. In settings with limited access to histopathological services, understanding age-, sex-, and site-based distribution patterns may support risk-based screening strategies. Moreover, through reanalyzing archival biopsy data using standardized diagnostic criteria from the 2022 WHO classification and applying robust statistical methods, this study contributes to improved comparability across regions and time periods, thereby supporting future meta-analyses and epidemiological modeling. The resulting insights have broad applicability across the clinical, educational, and research domains.

To our knowledge, this is the largest single-center study to reclassify over 19,000 jaw cysts using the 2022 WHO criteria, offering an unprecedentedly consistent epidemiologic and clinicopathologic dataset spanning nearly 50 years. This standardization enhances comparability with future studies. However, the retrospective, single-institution design of our study might limit the applicability of our findings to broader populations and settings.

## Conclusions

This study analyzed 19,352 histologically confirmed jaw cysts, reclassified under the 2022 WHO system, to clarify their epidemiological and clinicopathological characteristics. Odontogenic cysts, particularly radicular and dentigerous cysts, were most common, with distinct demographic and anatomical distributions. Notable findings included the regional predominance of surgical ciliated cysts and the mandibular bias of several cyst types. Sex- and age-specific trends were evident across multiple diagnostic categories, underscoring the relevance of demographic factors in clinical assessment. These observations reinforce the value of standardized classification systems and the clinical importance of site- and age-specific diagnostic awareness when evaluating jaw radiolucencies and forming differential diagnoses. This dataset offers a stable reference for future multicenter research and contributes to a global understanding of jaw cyst epidemiology.

## Data Availability

The datasets generated and/or analyzed during the current study are available from the corresponding author on reasonable request.

## Abbreviations

NOS: not otherwise specified
SD: standard deviation
WHO: World Health Organization
